# Correction: Genetic and morphological divergence in the warm-water planktonic foraminifera genus *Globigerinoides*

**DOI:** 10.1371/journal.pone.0326270

**Published:** 2025-06-11

**Authors:** Raphaël Morard, Angelina Füllberg, Geert-Jan A. Brummer, Mattia Greco, Lukas Jonkers, André Wizemann, Agnes K. M. Weiner, Kate Darling, Michael Siccha, Ronan Ledevin, Hiroshi Kitazato, Thibault de Garidel-Thoron, Colomban de Vargas, Michal Kucera

The Nomenclatural acts subsection is missing from the Methods. The Nomenclatural acts subsection should read as follows: The electronic edition of this article conforms to the requirements of the amended International Code of Zoological Nomenclature, and hence the new name contained herein is available under that Code from the electronic edition of this article. This published work and the nomenclatural act it contains have been registered in ZooBank, the online registration system for the ICZN. The ZooBank LSIDs (Life Science Identifiers) can be resolved and the associated information viewed through any standard web browser by appending the LSID to the prefix “http://zoobank.org/”. The LSID for this publication is: urn:lsid:zoobank.org:pub:45CB0650-1E70-473E-990C-3B337C481812. The electronic edition of this work was published in a journal with an ISSN, and has been archived and is available from the following digital repositories: PubMed Central PMC6894840).

There is an error in the ninth line of the Systematic section. The correct ninth line is: Subspecies *Globigerinoides ruber albus* n. subsp. urn:lsid:zoobank.org:act:6F0D7E68-CD65-43B1-8995-3F5719A105ED.
