# Supplementary figures and images for: Genetic and morphological divergence in the warm-water planktonic foraminifera genus Globigerinoides
Source: PLoS One. 2019 Dec 5;14(12):e0225246. doi: 10.1371/journal.pone.0225246 (PMC6894840; doi:10.1371/journal.pone.0225246)

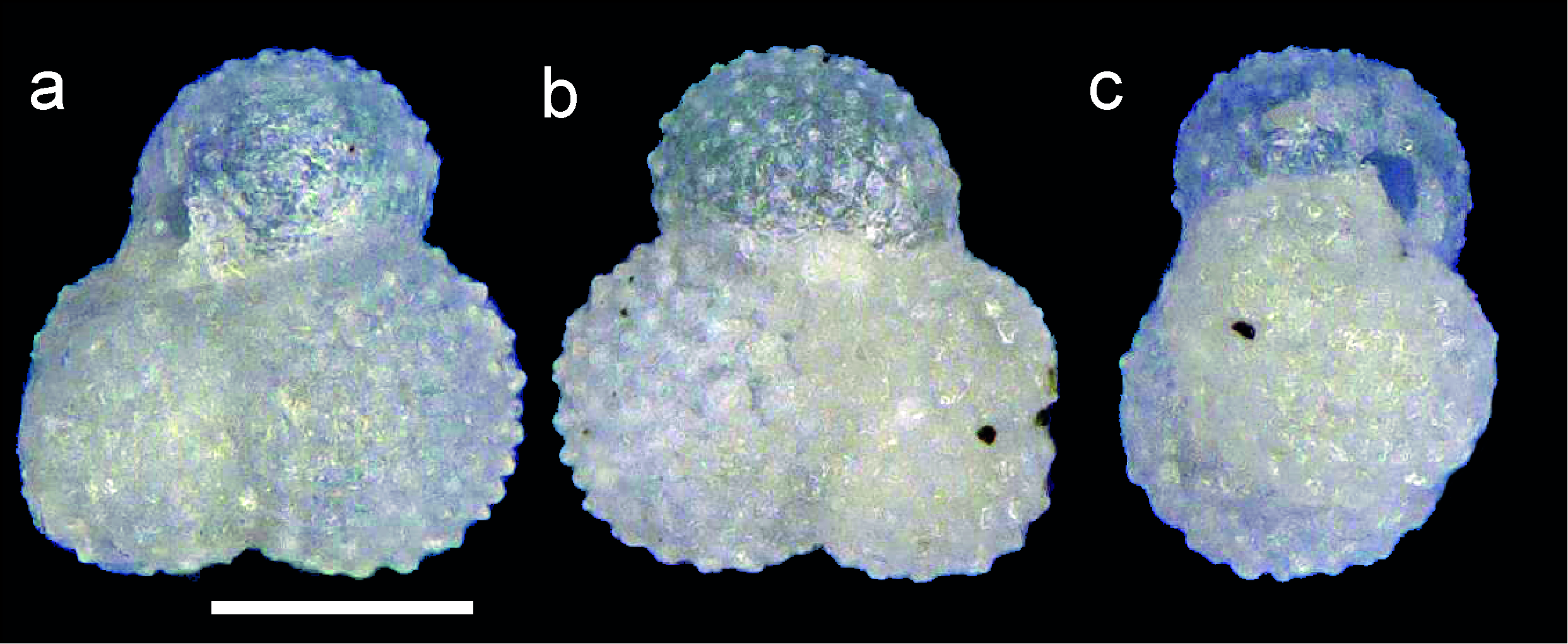

Supplement: S1 Fig — (a) Umbilical (b) spiral (c) lateral views. The scale bar represents 100 μm. (TIF) [file pone.0225246.s001.tif]

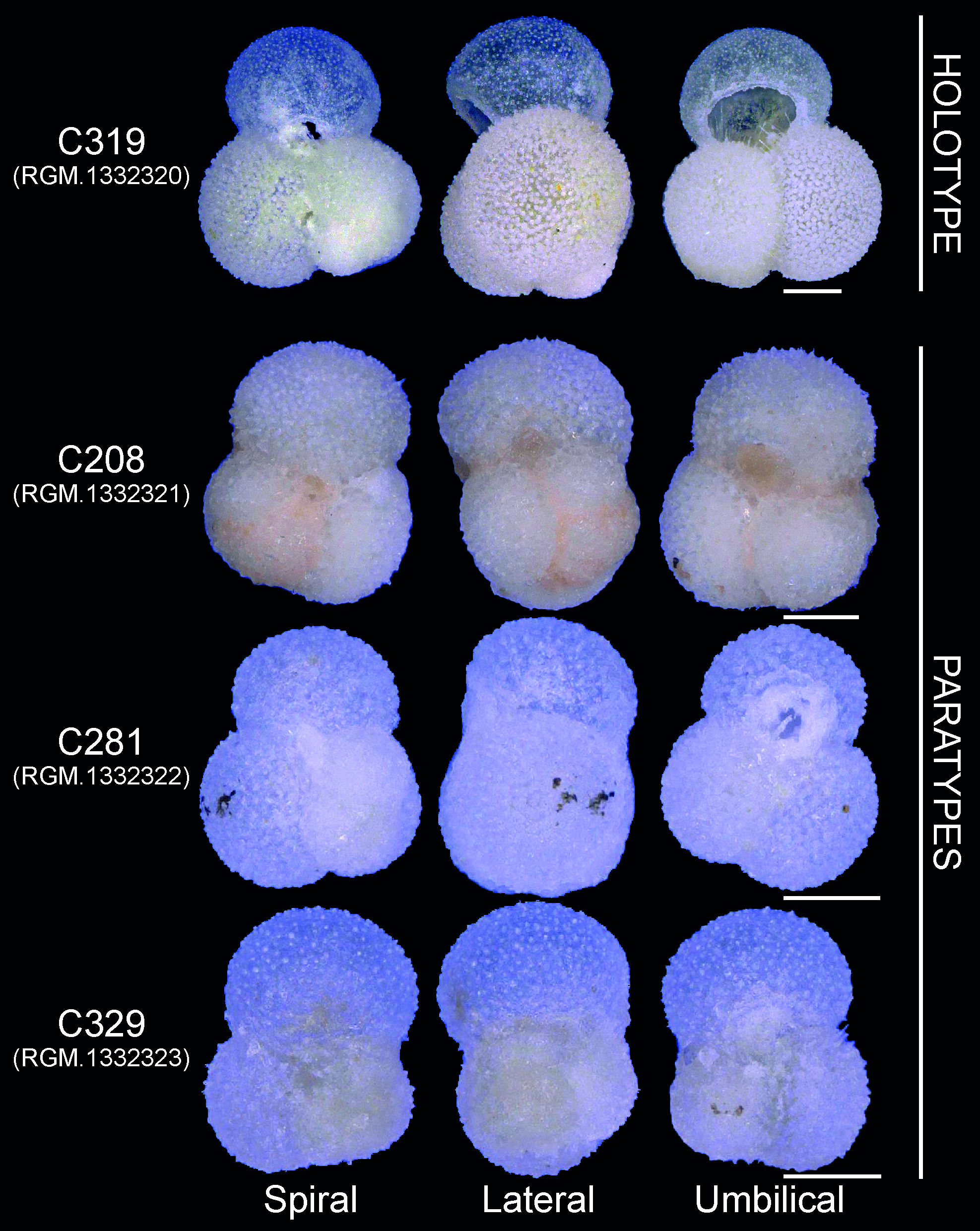

Supplement: S2 Fig — The archiving museum numbers at the Naturalis Biodiversity Center, Leiden, The Netherlands are provided below the voucher of the specimens. The scale bar represents 100 μm. (TIF) [file pone.0225246.s002.tif]
