# Supplementary material for: Genetic and morphological divergence in the warm-water planktonic foraminifera genus Globigerinoides
Source: PLoS One. 2019 Dec 5;14(12):e0225246. doi: 10.1371/journal.pone.0225246 (PMC6894840; doi:10.1371/journal.pone.0225246)
Supplement: Attachment — Submitted filename: Response to Reviewers.docx [file pone.0225246.s007.docx]

In the following, the comments of the reviewers are indicated by these symbols ***…*** and our responses are indicated by these symbols >>> …. <<<. We specify when necessary the page number of the changes we have made in the modified version of the manuscript with track changes.

*****Reviewer #1: I appreciate the detailed author’s reply to my first review. Whereas I agree to most of the author's points, I still have a couple of points to be addressed before publication of the manuscript:**

**Line 522, and following: Please check again, and change “Indo-Pacific” to “Indian and Pacific Oceans”*****

>>> We have made the changes following the reviewer’s request. Lines 522, 524, 533, 536, 538-539.<<<

**Line 587: carbon isotopes and trace elements in the calcite shell, better change to “calcareous” shell…**

>>> We have made the modification. Line 588.<<<

**Lines 653-654: “The roundness of the chambers of G. ruber remains stable throughout its ontogeny (Fig 10).”—I still don’t agree to this statement and the following discussion on neoteny. Juvenile G. ruber have a rather compressed bean shape, which changes into a more spherical shape in the neanic an adult stage. While the data and images (poor quality in my copy) provided here may not show the change in shape, this change in shape (visible also in the development of volume) is still the case. The authors may ask their senior co-author G.-J. Brummer, who may know best.**

>>> We are here describing the Raupian parameters that are descriptors of shape within the *Globigerinoides* genus. As it stands, such parameters are over-simplifying the complexity of a shape but have the merit to make the comparison between species easier, e.g. with numbers and not words. We agree that the evolution of the shape of *G. ruber* could be described following the reviewer’s words, but all the species have been subjected to the same analysis and we cannot help that *G. ruber* is the species displaying the least variation of the shape (Descriptor S) during its ontogeny. However, we agree with the reviewer that our formulation might not reflect other aspects of ontogenetic morphology and we have modified the statement in order to tone down this assertion. Lines 654-656.

Regarding the discussion on neoteny following the statement, we believe that we are entitled to express our ideas and interpretation of the patterns we observe even if the reviewer does not agree with us. The change in the pace of volume increase observed with *G. ruber* actually supports the idea that *G. ruber* follows a neotenic ontogenetic trajectory. Neoteny is defined as the retention of juvenile features (round chambers) in the adult without a reduction of the size. The increase of the size of the chamber mentioned by the reviewer may compensate for the fewer chambers in the analyzed specimen. As a result, this allowed the specimen to reach the typical adult size of *G. ruber*, similar to *G. elongatus* whilst *G. tenellus* which also has fewer chambers (but with similar chamber size to *G. elongatus*) is overall smaller.

We have had lengthy discussions on the question with all co-authors, among which G. -J Brummer and we all agreed on the content of the manuscript before submission, including the heterochrony and the relevance of using Raupian parameters to describe the development of members of the *Globigerinoides* genus. We consider that our manuscript is open to debatable interpretation and we are not here pretending that heterochrony is the definitive answer, only a potential explanation.<<<
